# Targeting mGluR2/3 Signaling With LY341495 Restores Dentate Gyrus Function and Cognitive Performance in a Male Mouse Model of Alzheimer's Disease

**DOI:** 10.1002/cns.70916

**Published:** 2026-05-15

**Authors:** Cui‐Ping Chen, Ting Zhang, Er‐Deng E, Tian‐Yang Xu, Qing Han, Xiang Gao, Jie Sun, Yi Huang, Jian‐Hong Yang, Xiao‐Qin Zhang

**Affiliations:** ^1^ Cerebrovascular Disease Center, Ningbo Key Laboratory of Nervous System and Brain Function The First Affiliated Hospital of Ningbo University Ningbo Zhejiang China; ^2^ Department of Pharmacology, Health Science Center Ningbo University Ningbo Zhejiang China; ^3^ Ningbo Key Laboratory of Nervous System and Brain Function, Department of Neurosurgery The First Affiliated Hospital of Ningbo University Ningbo Zhejiang China

**Keywords:** Alzheimer's disease, cognitive deficits, dentate gyrus, LY341495, metabotropic glutamate receptors

## Abstract

**Background:**

Aberrant metabotropic glutamate receptor 2/3 (mGluR2/3) signaling has been implicated in the synaptic and cognitive deficits observed in Alzheimer's disease (AD), yet the underlying regulatory mechanisms remain unclear. This study investigated the therapeutic potential of LY341495, a selective mGluR2/3 antagonist, in APP/PS1 transgenic mice, a widely used AD model.

**Methods:**

Male APP/PS1 mice were treated with the selective mGluR2/3 antagonist LY341495. Cognitive performance was evaluated using behavioral tests. Hippocampal dentate gyrus (DG) alterations were examined by immunohistochemistry and electrophysiology, including analyses of mGluR2/3 expression, excitatory synaptic activity, adult neurogenesis, calbindin expression, and amyloid‐β plaque burden.

**Results:**

APP/PS1 mice exhibited pathological upregulation of mGluR2/3 in the DG, accompanied by altered presynaptic glutamatergic transmission, reduced neurogenesis, decreased calbindin expression, and deficits in recognition and spatial memory. LY341495 treatment attenuated the aberrant mGluR2/3 upregulation, enhanced excitatory synaptic activity, and improved calbindin levels and neurogenesis in the DG. Importantly, these changes were associated with significant reductions in DG amyloid‐β plaque burden and marked improvements in cognitive performance.

**Conclusions:**

This study highlights the novelty of linking mGluR2/3 inhibition to the restoration of calcium‐buffering capacity, as reflected by calbindin expression and neurogenesis, processes critical for DG plasticity and resilience. These findings underscore the therapeutic potential of LY341495 as a novel intervention targeting mGluR2/3 signaling in AD.

## Graphical abstract

LY341495 inhibits mGluR2/3 signaling and improves dentate gyrus function in APP/PS1 mice. Treatment enhances excitatory synaptic activity, increases calbindin expression and neurogenesis, and reduces amyloid‐β plaque burden. These changes are associated with improved cognitive performance.

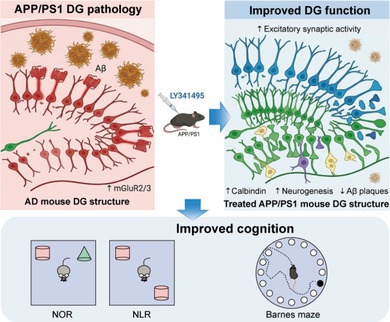

## 1 Introduction

Alzheimer's disease (AD) is a progressive neurodegenerative disorder characterized by memory impairments and synaptic dysfunction. A hallmark feature of AD is the accumulation of amyloid‐β (Aβ) plaques, which are closely associated with neuronal damage and disrupted synaptic plasticity, particularly in the hippocampus, a brain region critically involved in learning and memory [1]. Within the hippocampus, the dentate gyrus (DG) plays a central role in adult neurogenesis and synaptic integration, both of which are markedly impaired in ad [2, 3]. Emerging evidence highlights the importance of calcium‐binding proteins, such as calbindin‐D28k, in maintaining intracellular calcium balance and supporting neuronal function. In AD, calbindin levels are significantly reduced, particularly in the DG, contributing to calcium dysregulation, synaptic dysfunction, and cognitive decline [4]. Another critical mechanism underlying synaptic dysfunction in AD involves dysregulation of glutamatergic signaling, particularly through the metabotropic glutamate receptor (mGluR) system [5]. However, the precise relationship among calbindin, neurogenesis, and mGluR signaling in the hippocampus remains unclear. This study explores whether targeting mGluR2/3, key regulators of glutamate transmission, can improve synaptic and cognitive function in the context of AD.

mGluR2 and mGluR3 are group II metabotropic glutamate receptors that function as inhibitory modulators of glutamate release at presynaptic terminals. These receptors are involved in regulating excitatory neurotransmission, maintaining synaptic homeostasis, and protecting neurons from excitotoxicity [6]. LY341495, a selective mGluR2/3 antagonist, has been shown to enhance glutamate release by blocking the inhibitory effects of mGluR2/3 on presynaptic terminals [7]. Previous studies suggest that LY341495 can improve cognitive performance and synaptic transmission in preclinical models of neurodevelopmental disorders by modulating glutamatergic signaling [8]. In addition, the mGlu2 receptor positive allosteric modulator LY566332 has been reported recently to amplify Aβ‐induced neurodegeneration, and this effect was prevented by LY341495 administration [9]. Moreover, LY341495 was found to block the sustained accumulation of Aβ_42_ and prevent the degradation of APP C‐terminal fragments [10]. However, the precise mechanisms underlying the therapeutic effects of LY341495 remain insufficiently understood. Specifically, its influence on calcium‐buffering proteins such as calbindin, as well as its effects on adult neurogenesis and synaptic plasticity, require further investigation. These processes are pivotal for hippocampal function and resilience, particularly in the context of AD.

In this study, we investigated the effects of LY341495 on hippocampal function in male APP/PS1 mice, a widely used transgenic model of AD. We confirmed reduced calbindin expression, impaired neurogenesis, and synaptic dysfunction in the DG of APP/PS1 mice. LY341495 treatment effectively improved recognition and spatial memory, reduced hippocampal plaque load, restored calbindin levels, enhanced neurogenesis, and increased excitatory synaptic activity, with no notable effects in healthy WT controls. These results support the hypothesis that LY341495 mitigates hippocampal DG dysfunction in AD by targeting mGluR2/3 signaling. By restoring calbindin expression, a key calcium‐buffering protein, together with improvements in neurogenesis and synaptic transmission, LY341495 may contribute to the functional recovery of DG circuitry. This study highlights the critical role of mGluR2/3 signaling in AD pathophysiology and its potential as a therapeutic target.

## 2 Materials and Methods

### 2.1 Animals

Male APP/PS1 transgenic mice (Jax, 034829) and their wild‐type (WT) littermates (6 months, male) were obtained from Hangzhou Ziyuan Laboratory Animal Technology Co. Ltd. All mice were housed under standard laboratory conditions, maintained on a 12‐h light/dark cycle at 22°C ± 1°C with 50% ± 10% relative humidity, and provided ad libitum access to food and water. Drug administration was initiated between Days 6–12, followed by behavioral testing conducted on Days 13–19 and 26–30. Mice were randomly assigned to one of four groups based on genotype and treatment: (1) WT + Vehicle, (2) WT + LY341495, (3) APP/PS1 + Vehicle, and (4) APP/PS1 + LY341495.

All experimental procedures were performed in accordance with the National Institutes of Health Guide for the Care and Use of Laboratory Animals, and were approved by the Animal Care and Use Committees of Ningbo University (NBU20240354, December 2024), China.

### 2.2 Drug Administration

Mice in the LY341495‐treated groups (WT + LY341495 and APP/PS1 + LY341495) received daily intraperitoneal injections of LY341495 (MCE #HY 70059, 1.0 mg/kg) for 7 consecutive days. Control groups (WT + Vehicle and APP/PS1 + Vehicle) were administered equivalent volumes of saline following the same protocol. The dosage of LY341495 was selected based on our previous study, which demonstrated its potential to improve cognitive function in mouse models of neurodevelopmental disorders [8].

### 2.3 Behavioral Tests

**FIGURE 1:**
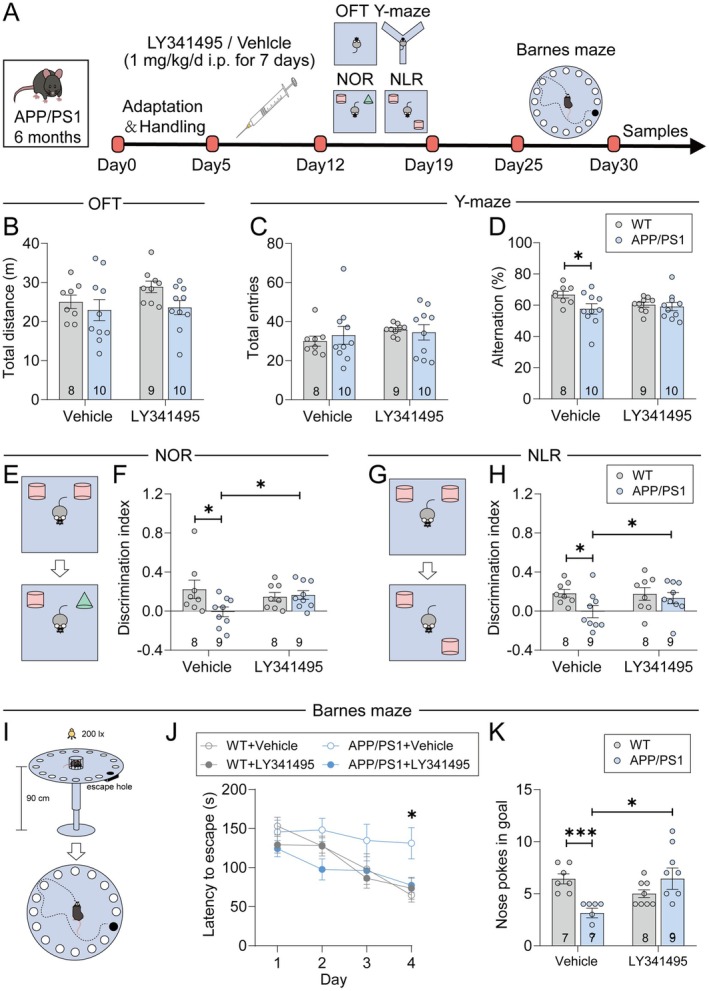
LY341495 improved cognitive performance in APP/PS1 mice. (A) Experimental timeline showing treatment and behavioral tests, including the open field test (OFT), Y‐maze, novel object recognition (NOR), novel location recognition (NLR), and Barnes maze. (B–D) Results of the OFT and Y‐maze. (B) Total distance traveled in the OFT. (C) Total arm entries in the Y‐maze. (D) Percentage of alternations in the Y‐maze. (E–H) NOR and NLR tests. (F) Discrimination index for NOR, showing a significant improvement in APP/PS1 mice treated with LY341495 compared to the vehicle group (*p < 0.05). (H) Discrimination index for NLR, showing a significant improvement in APP/PS1 mice treated with LY341495 (*p < 0.05). (I–K) Barnes maze performance. (I) Schematic of the Barnes maze. (J) Escape latency across 4 training days, showing significantly reduced latency in APP/PS1 mice treated with LY341495 compared to the vehicle group on Day 4 (*p < 0.05). (K) Nose pokes in the target hole during the probe test, showing significant improvements in LY341495‐treated APP/PS1 mice (*p < 0.05, ***p < 0.001). Data are presented as mean ± SEM. Statistical significance was determined using two‐way ANOVA or unpaired *t*‐tests where appropriate. N represents the number of mice per group. Exact sample sizes are indicated in each figure panel.

Behavioral experiments were conducted between 8:30 a.m. and 8:30 p.m. Prior to the testing phase, mice underwent a 5‐day acclimatization period, during which they were habituated to the experimental room and handler through daily 5‐min sessions. Following acclimatization, mice received daily intraperitoneal injections of either LY341495 or vehicle for 7 consecutive days. Behavioral testing commenced 24 h after the final injection and was performed in the following sequence: open field test, Y‐maze test, novel location recognition (NLR), and novel object recognition (NOR). After a 6‐day recovery period, the Barnes maze test was conducted to assess long‐term spatial learning and memory (Figure 1A). All behavioral testing and data analyses were performed by experimenters blinded to genotype and treatment conditions.

#### 2.3.1 Open Field Test

The open field test was used to assess locomotor activity. The apparatus consisted of a white, open‐top acrylic chamber (40 × 40 × 40 cm). Before each trial, the chamber was cleaned with 75% alcohol and thoroughly dried. Mice were individually placed at the center of the chamber and allowed to explore freely for 10 min. The entire session was recorded using an infrared camera (Xiaoyi, China), and movement data were analyzed with ANY‐maze software (Stoelting, UK).

#### 2.3.2 Y‐Maze Test

The Y‐maze test was used to evaluate short‐term spatial working memory. The maze consisted of three identical arms (40 × 10 × 15 cm) arranged at 120° angles. Mice were placed at the center and allowed to explore freely for 8 min. Two main parameters were recorded: (1) total arm entries, defined as the mouse fully entering an arm with all four paws, and (2) spontaneous alternation, defined as consecutive entries into all three arms without repetition. The spontaneous alternation rate was calculated using the formula: (total spontaneous alternations/(total arm entries −2)) × 100.

#### 2.3.3 NLR and NOR Tests

NLR and NOR tests were used to assess spatial and object recognition memory, respectively. Both tests consisted of three phases: habituation, training, and testing. Prior to training, mice were acclimated to an open‐field box (25 × 25 × 25 cm, with one interior wall marked) for 10 min per day over three consecutive days.

##### 2.3.3.1 NLR Test

Twenty‐four hours after acclimation, mice were exposed to two identical objects during a 10‐min training session. One hour later, during the testing phase, one object was moved to a diagonal position, and mice were allowed to explore the objects for 5 min. Object exploration was defined as a mouse's nose touching or orienting toward an object within 2 cm. The investigation time during the 5‐min session was recorded, and the NLR index was calculated as follows: NLR Index = (novel location investigation time − familiar location investigation time) / (novel location investigation time + familiar location investigation time).

##### 2.3.3.2 NOR Test

The NOR test followed a similar protocol to the NLR test during the first 4 days. However, on the fifth day, instead of moving an object, one of the identical objects was replaced with a novel object of a different shape but identical material. Mice explored the objects for 5 min, and the NOR index was calculated using the formula: NOR Index = (novel object investigation time − familiar object investigation time) / (novel object investigation time + familiar object investigation time).

To ensure reliable results, the apparatus was cleaned with 75% ethanol and thoroughly dried after each trial.

#### 2.3.4 Barnes Maze Test

The Barnes maze is a widely used behavioral paradigm for assessing spatial learning and memory [11]. The apparatus consisted of a white circular platform (90 cm in diameter) with 20 evenly spaced holes (5 cm in diameter) positioned 4 cm from the edge. The maze was elevated 90 cm above the floor and illuminated by a 200‐lx overhead light, serving as a mild aversive stimulus. Visual cues were placed on the laboratory walls within 50 cm of the maze. The experiment included 4 training days followed by 1 probe test day. During training, a dark Plexiglas escape box (10 × 10 × 6 cm) lined with clean bedding was placed beneath a designated hole, providing shelter from the light. Mice were trained to locate the escape box over four daily trials to learn its position. Each trial began with a 30‐s habituation period in which the mouse was placed inside a cylindrical metal cage at the platform's center. The cage was then gently lifted, and the overhead light was turned on, allowing the mouse to explore the maze freely for up to 180 s to locate the escape box. If the mouse failed to find the box within the allotted time, it was guided to the escape box by the experimenter. After reaching the escape box, the mouse remained inside for 30 s before being returned to its home cage. Inter‐trial intervals lasted 15–25 min. Escape latency, defined as the time taken to locate the escape box after leaving the start cage, was recorded for all trials. Latency was recorded as 180 s if the mouse failed to locate the escape box. To prevent spatial bias, the platform was rotated 90° after each trial during training, excluding the probe test. On Day 5 (probe test), the escape box was removed, and each mouse was allowed to explore the maze for 90 s. Escape latency and the number of nose pokes at the goal hole were recorded manually [12]. The platform was cleaned with 70% ethanol and thoroughly dried before each session to eliminate odor cues.

### 2.4 Electrophysiology

Brain slices containing the hippocampus were prepared from 6‐ to 7‐month‐old mice. Mice were deeply anesthetized with 50 mg/kg pentobarbital sodium, decapitated, and the brain was quickly transferred to a chamber filled with ice‐cold cutting solution (in mM: 75 sucrose, 87 NaCl, 3 KCl, 1.5 CaCl_2_, 1.3 MgCl_2_, 1 NaH_2_PO_4_, 26 NaHCO_3_, 20 glucose) equilibrated with 95% O_2_–5% CO_2_. Coronal brain slices (220 μm thick) were prepared using a vibratome (Leica VT1200S, Leica Microsystems, Germany) and incubated in oxygenated artificial cerebrospinal fluid (aCSF; in mM: 124 NaCl, 3 KCl, 1 NaH_2_PO_4_, 1.3 MgCl_2_, 2 CaCl_2_, 26 NaHCO_3_, 20 glucose; 295–305 mOsm, 95% O_2_–5% CO_2_) at 32°C for 30 min. Slices were then maintained at room temperature (20°C–22°C) for at least 1 h before recordings. During patch‐clamp recordings, slices were transferred to a recording chamber and perfused with oxygenated aCSF (32°C) at a flow rate of 2 mL/min.

Standard whole‐cell recordings were performed on dentate granule cells (GCs) in hippocampal slices using a Multiclamp 700B amplifier and Digidata 1550B digitizer (Molecular Devices, CA, USA). GCs were identified under infrared differential interference contrast (IR‐DIC) microscopy based on their location and morphology. Patch pipettes, with a resistance of 1.5–2 MΩ, were pulled from thin‐wall borosilicate glass (TW150‐3, WPI) using a vertical puller (PC‐10, Narishige). Recordings were made using a Heka EPC 10 amplifier (Heka Elektronik), with careful adjustments for fast and slow capacitance and series resistance compensation. Holding potentials were corrected for liquid junction potential, and recordings with series resistance changes exceeding 20% were excluded. Data were filtered at 1 kHz and digitized at 20 kHz. Spontaneous excitatory postsynaptic currents (sEPSCs) were recorded at −70 mV using a cesium‐based internal solution (in mM: 110 cesium methylsulfate, 15 CsCl, 4 Mg‐ATP, 0.3 Na₂‐GTP, 0.5 EGTA, 10 HEPES, 4 QX‐314, 5 phosphocreatine‐Na₂; pH 7.2–7.4, 270–280 mOsm) and in the presence of picrotoxin (100 μM) to block GABA_A receptor‐mediated inhibitory currents. The sEPSCs with LY341495 (1 μM; MCE #HY‐70059) incubation were recorded for APP/PS1 mice. Evoked EPSCs (eEPSCs) were recorded by electrically stimulating presynaptic axons with a bipolar stimulating electrode (FHC Inc., Bowdoin, ME) positioned 200 μm from the recorded GC soma. Constant current pulses (25, 50, and 100 μs duration, 0.2 Hz frequency) were delivered, and eEPSCs were recorded at −70 mV using the same cesium‐based internal solution and picrotoxin to inhibit inhibitory currents. Offline analysis was conducted using AxoGraph 4.6 software (Axon Instruments, Union City, CA, USA).

### 2.5 Immunostaining and Quantification

Mice were anesthetized with sodium pentobarbital (50 mg/kg, i.p.) and perfused with PBS. Brains were immediately isolated and fixed in 4% paraformaldehyde (PFA) at 4°C overnight. After 24 h of fixation, brains were transferred to 30% sucrose at 4°C for 3 days until fully dehydrated. Coronal sections (30 μm thick) were prepared using a Leica cryostat. For immunohistochemical staining, after quenching endogenous peroxidase activity by incubation with 3% H_2_O_2_ in methanol, sections were incubated with the following primary antibodies (6E10, 1:500, 803,001, BioLegend; Calbindin, 1:500, sc‐365,360, Santa Cruz Biotechnology; mGluR2/3, 1:500, ab150387, Abcam; DCX, 1:500, 4604S, CST). Afterwards, the slices were incubated with SABC solution at 37°C for 30 min and finally incubated with ABC Vector Elite kit containing avidin and biotin (SA1022, BOSTER Biological Technology Co. Ltd. China).

For quantification of DCX^+^ cells, three brain sections spanning the DG between bregma −1.46 mm and −3.16 mm were analyzed for each animal. DCX^+^ cells in the DG were quantified by capturing images under a 20× objective using a fluorescent microscope (Olympus BX51, Japan). For the quantification of mGluR2/3^+^ and calbindin^+^ levels, the integrated optical density (IOD) was measured in two areas: the molecular layer of the DG and the stratum radiatum of the CA1 region. Calbindin expression was calculated as the ratio of IOD in the molecular layer of the DG to that in the stratum radiatum of the CA1 region. Similarly, mGluR2/3 expression was determined as the ratio of IOD in the lateral (LMol) and middle (Mol) molecular layers of the DG to that in the CA1 stratum radiatum. Plaque load was quantified as the percentage of hippocampal area covered by 6E10‐positive immunoreactivity. For each mouse, three coronal sections were analyzed, and the average of individual measurements was used to calculate group means. All immunostaining analyses were conducted blindly to ensure objectivity.

### 2.6 Statistical Analysis

Data were analyzed using GraphPad Prism 10.0 (GraphPad software, San Diego, CA, USA). Two‐way ANOVA was carried out to test the interaction between the LY341495 treatment and APP/PS1 genotypes. Statistical significance between genotypes (WT vs. APP/PS1) under the same or different treatments (Vehicle vs. LY341495) within the same genotype was indicated by an asterisk (*), as determined by unpaired *t*‐tests. All data presented as mean ± SEM. Only values with *p* < 0.05 were accepted as statistically significant. Sample sizes (n/N) for each experiment are clearly indicated in the corresponding figure legends.

## 3 Results

### 3.1 LY341495 Improved Recognition and Spatial Memory in APP/PS1 Mice

To investigate the effects of LY341495 on cognitive function, we conducted a series of behavioral tests, starting with motor and spontaneous exploratory activity assessments to exclude potential confounding factors. The open field test (OFT) showed no significant differences in locomotion activity among all groups (Figure 1B), ensuring comparable motor function. Similarly, Y‐maze analysis revealed no significant differences in the total number of arm entries across groups (Figure 1C), confirming that exploratory behaviors were unaffected by genotype or treatment. However, APP/PS1 mice exhibited a lower spontaneous alternation percentage compared to WT mice (Figure 1D), reflecting impaired short‐term spatial working memory. Notably, LY341495 treatment did not significantly improve the spontaneous alternation percentage in APP/PS1 mice (Figure 1D), suggesting that the treatment had no measurable effect on short‐term working memory in this model.

We next evaluated recognition memory using the novel object recognition (NOR) and novel location recognition (NLR) tests. APP/PS1 mice showed significant impairments in both tasks, as indicated by a reduced discrimination index compared to WT controls (Figure 1E–H). LY341495 treatment selectively improved recognition memory in APP/PS1 mice, evidenced by a significant increase in the NOR and NLR discrimination indices (Figure 1F,H). These findings indicate that APP/PS1 mice exhibit deficits in object and spatial recognition memory, which are ameliorated by LY341495 treatment.

To further assess long‐term spatial learning and memory, we employed the Barnes maze, a higher‐complexity behavioral paradigm. During the four‐day training phase, escape latency progressively decreased in WT mice, reflecting improved spatial learning. By Day 4, LY341495‐treated APP/PS1 mice also demonstrated significantly shortened escape latencies compared to their vehicle‐treated counterparts, demonstrating enhanced spatial learning. Notably, no significant effects of LY341495 were observed in WT controls. On the probe test (Day 5), APP/PS1 mice treated with vehicle exhibited fewer nose pokes in the target hole, consistent with impaired spatial memory [11, 13]. Remarkably, LY341495 treatment fully rescued this deficit in APP/PS1 mice, restoring nose poke counts to levels comparable to those of WT controls (Figure 1I–K). These results highlight the ability of LY341495 to effectively improve both spatial learning and memory in APP/PS1 mice.

### 3.2 LY341495 Reduced Hippocampal DG Plaque Load in APP/PS1 Mice

**FIGURE 2:**
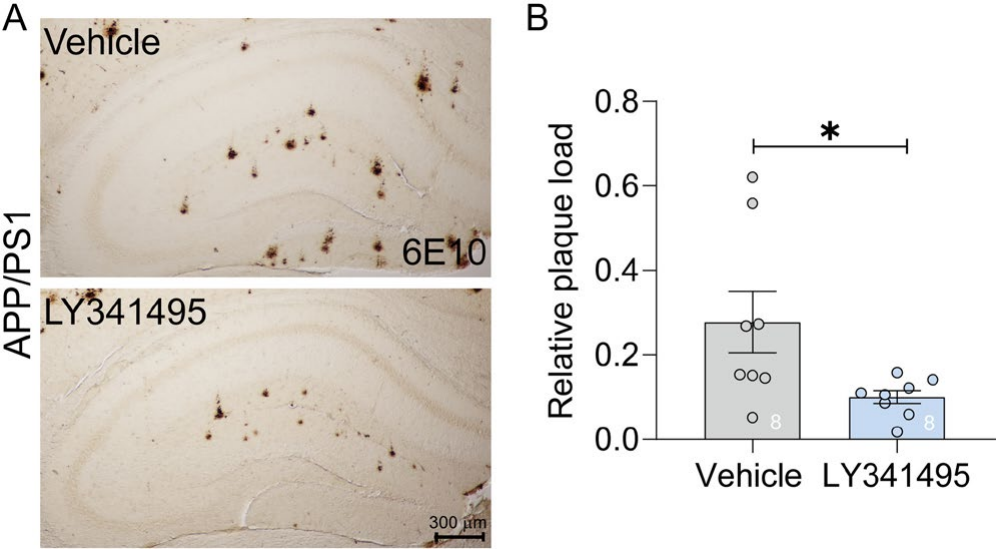
LY341495 reduced hippocampal plaque load in APP/PS1 mice. (A) Representative immunohistochemical images of hippocampal sections stained with 6E10 antibody from APP/PS1 mice treated with either vehicle (top) or LY341495 (bottom). Scale bar: 300 μm. (B) Quantification of relative plaque load in the hippocampus. LY341495 treatment significantly reduced plaque burden compared to the vehicle group (mean ± SEM, *p < 0.05, unpaired *t*‐test). N represents the number of mice per group. Exact sample sizes are indicated in each figure panel.

Following the completion of all behavioral tests, DAB staining was performed to assess Aβ plaque load in APP/PS1 mice. The results revealed that a one‐week LY341495 treatment regimen administered to 6‐month‐old APP/PS1 mice significantly attenuated the progression of Aβ plaques by the age of 7 months. Aβ plaques in the hippocampus were predominantly localized to the DG (Figure 2A). Notably, LY341495 treatment markedly reduced plaque burden in these regions, suggesting that the drug effectively mitigates Aβ accumulation in the hippocampus (Figure 2B). These findings demonstrate the potential of LY341495 to delay hippocampal DG Aβ pathology in APP/PS1 mice.

### 3.3 LY341495 Attenuated Pathological Upregulation of mGluR2/3 in APP/PS1 Mice

**FIGURE 3:**
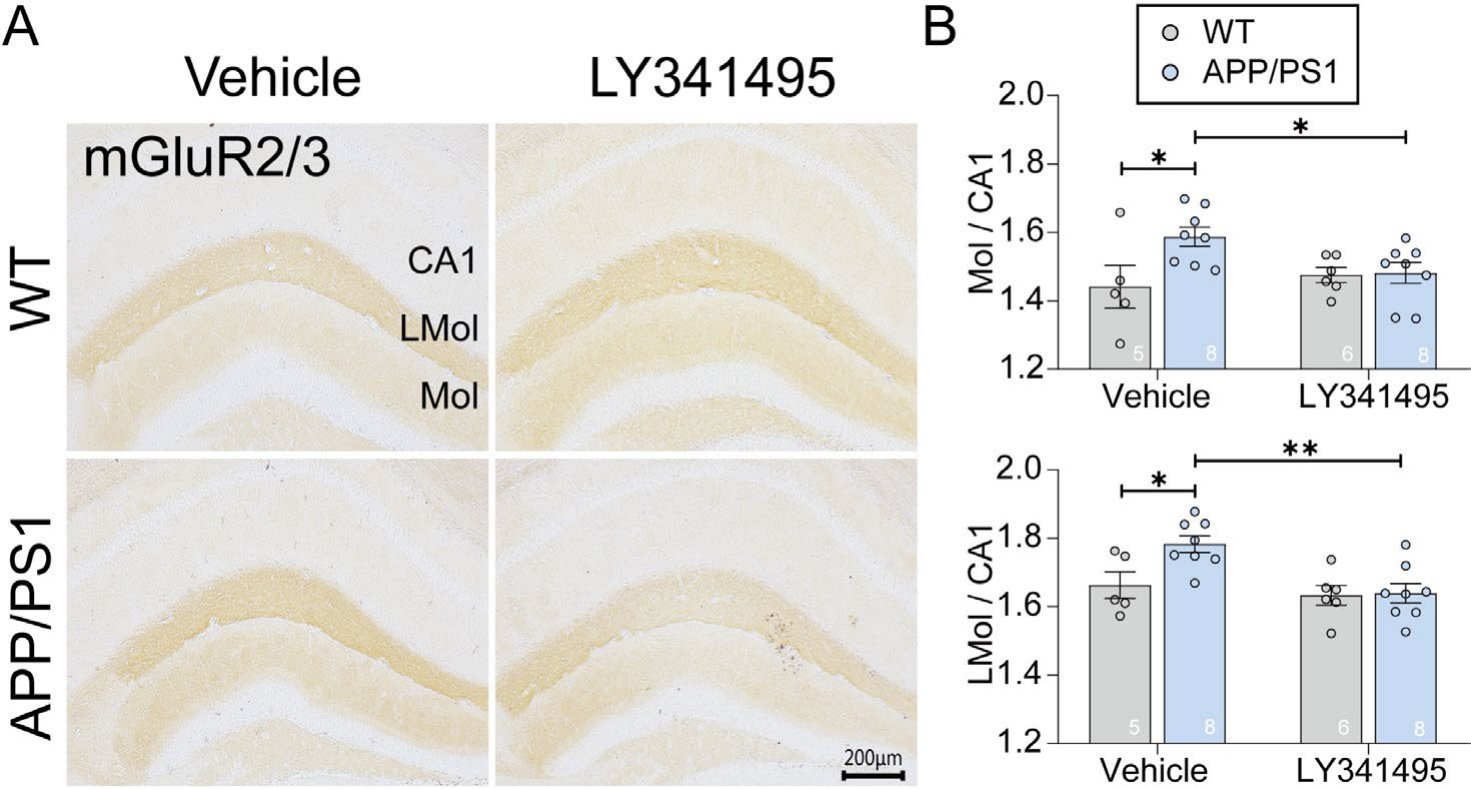
LY341495 normalized mGluR2/3 levels in the DG of APP/PS1 mice. (A) Representative immunohistochemical images showing mGluR2/3 staining in the lateral (LMol) and middle (Mol) molecular layers of the DG and CA1 region in WT and APP/PS1 mice treated with vehicle or LY341495. Scale bar: 200 μm. (B) Quantification of mGluR2/3 expression levels in the hippocampus. The ratios of mGluR2/3 intensity in the Mol to CA1 (top) and in the LMol to CA1 (bottom) were significantly reduced in vehicle‐treated APP/PS1 mice compared to WT controls (*p < 0.05). LY341495 treatment significantly increased mGluR2/3 expression in APP/PS1 mice (**p < 0.01), restoring levels comparable to WT mice. Data are presented as mean ± SEM. Statistical significance was determined using two‐way ANOVA or unpaired *t*‐tests where appropriate. N represents the number of mice per group. Exact sample sizes are indicated in each figure panel.

Metabotropic glutamate receptors 2 and 3 (mGluR2/3) play critical roles in brain function and are targeted by the antagonist LY341495. Our analysis revealed that 7‐month‐old APP/PS1 mice exhibited significantly elevated mGluR2/3 levels, predominantly localized to plaque‐rich regions of the hippocampus, particularly in the lateral (LMol) and middle (Mol) molecular layers of the DG (Figure 3A,B). This pathological upregulation is consistent with amyloid plaque deposition driving aberrant receptor expression. These findings suggest that amyloid plaques are closely associated with the aberrant expression of mGluR2/3 receptors in hippocampal regions, potentially contributing to synaptic dysfunction and cognitive deficits. Importantly, LY341495 treatment significantly attenuated the pathological upregulation of mGluR2/3 immunoreactivity in APP/PS1 mice (Figure 3A,B), bringing receptor levels closer to those observed in WT controls. We note that this effect is unlikely to reflect direct suppression of receptor expression by LY341495, but rather may represent a secondary consequence of reduced Aβ burden and restored synaptic activity.

### 3.4 LY341495 Restored Calbind in Levels in the DG of APP/PS1 Mice

**FIGURE 4:**
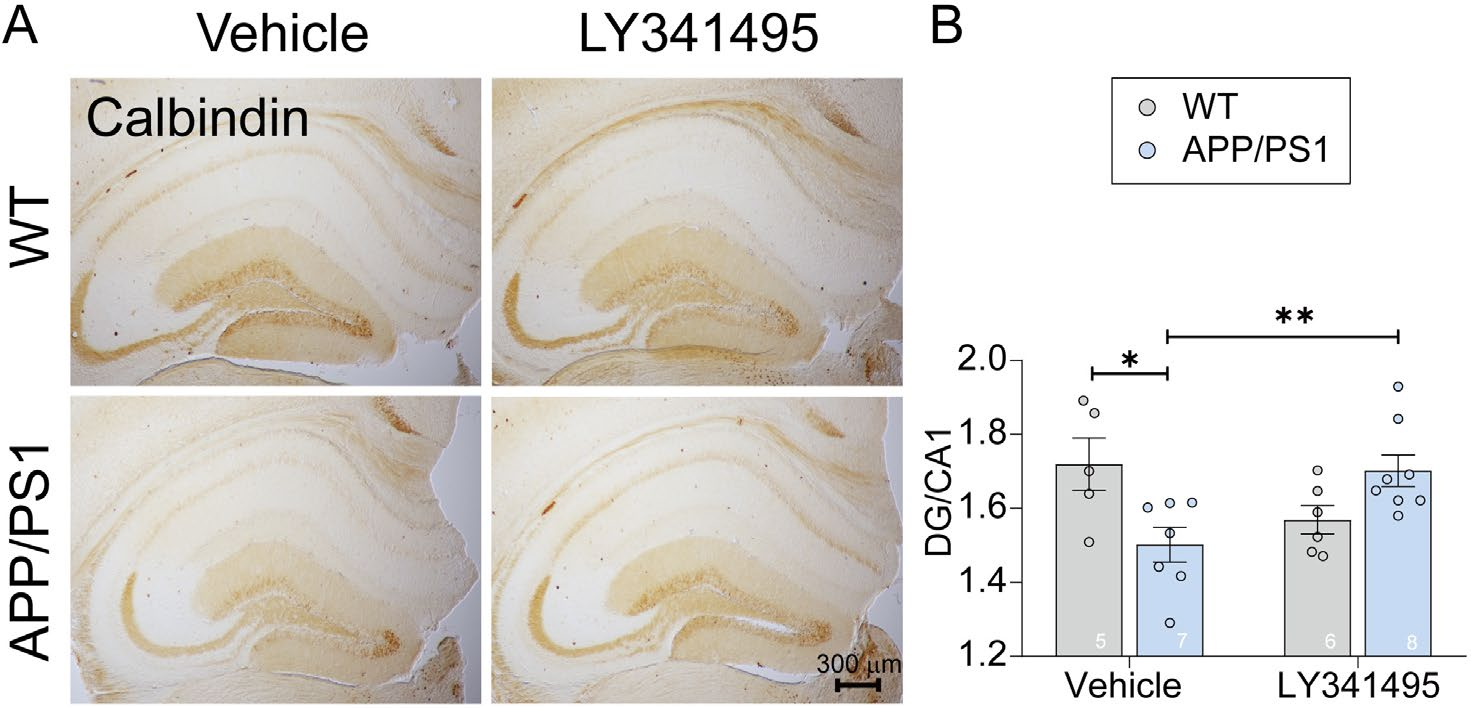
LY341495 restored calbindin levels in the DG of APP/PS1 mice. (A) Representative immunohistochemical images showing calbindin staining in the DG and CA1 region of WT and APP/PS1 mice treated with vehicle or LY341495. Scale bar: 300 μm. (B) Quantification of calbindin expression levels. The ratio of calbindin intensity in the DG to the CA1 region was significantly reduced in vehicle‐treated APP/PS1 mice compared to WT controls (*p < 0.05). LY341495 treatment significantly increased calbindin expression in APP/PS1 mice, restoring it to levels comparable to WT controls (**p < 0.01). Data are presented as mean ± SEM. Statistical significance was determined using two‐way ANOVA or unpaired *t*‐tests where appropriate. N represents the number of mice per group. Exact sample sizes are indicated in each figure panel.

Studies have shown that calbindin‐D28k levels are reduced in the DG of AD patients and in AD mouse models. This reduction may impair calcium‐buffering capacity, potentially contributing to neuronal dysfunction and cognitive impairments observed in ad [14, 15, 16]. To determine whether calbindin alterations contribute to the therapeutic effects of LY341495, we evaluated its expression in APP/PS1 mice following treatment. Consistent with our previous reports [13], calbindin levels were significantly reduced in the DG of APP/PS1 mice compared to WT controls (Figure 4A,B). LY341495 treatment effectively restored the calbindin levels in APP/PS1 mice, restoring it to levels comparable to WT controls (Figure 4A,B). Notably, calbindin expression was particularly diminished in the middle molecular layer (Mol) of the DG. LY341495 selectively increased calbindin levels in APP/PS1 mice in this region, without affecting expression in WT controls.

### 3.5 LY341495 Enhanced Hippocampal Neurogenesis in APP/PS1 Mice

**FIGURE 5:**
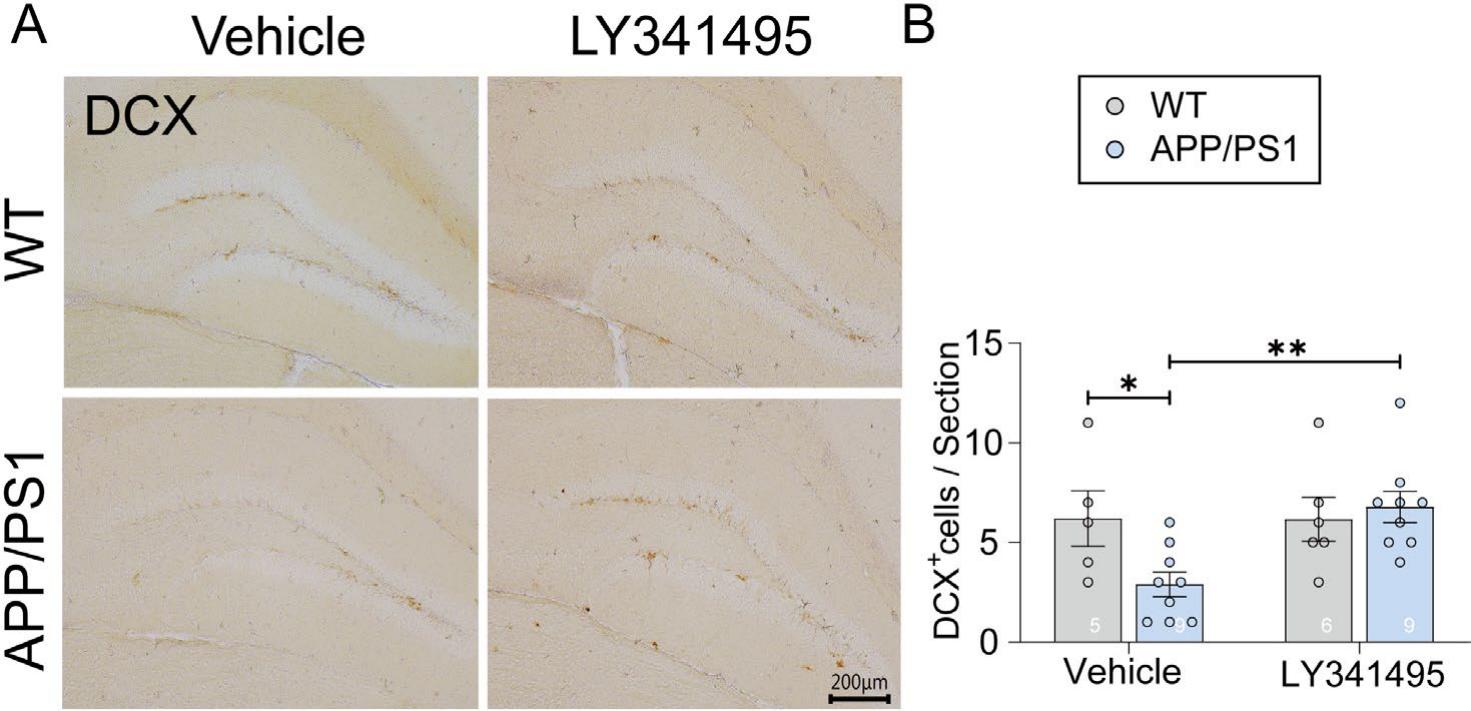
LY341495 enhanced neurogenesis in the DG of APP/PS1 mice. (A) Representative immunohistochemical images of DCX^+^ cells in the DG of WT and APP/PS1 mice treated with vehicle or LY341495. Scale bar: 200 μm. (B) Quantification of DCX^+^ cells per section in the DG. The number of DCX^+^ cells in the DG was significantly reduced in vehicle‐treated APP/PS1 mice compared to WT controls (*p < 0.05). LY341495 treatment significantly increased the number of DCX^+^ cells in APP/PS1 mice compared to the vehicle group (**p < 0.01). Data are presented as mean ± SEM. Statistical significance was determined using two‐way ANOVA or unpaired *t*‐tests where appropriate. N represents the number of mice per group. Exact sample sizes are indicated in each figure panel.

Furthermore, calcium‐binding proteins are known to play critical roles in neurogenesis and neuroprotection [17]. To evaluate the impact of LY341495 on hippocampal neurogenesis, we quantified doublecortin‐positive (DCX^+^) cells, a marker of immature neurons, in the DG. APP/PS1 mice exhibited a significant reduction in the number of DCX^+^ cells compared to WT controls, indicating impaired neurogenesis (Figure 5A,B). LY341495 treatment significantly increased the number of DCX^+^ cells in APP/PS1 mice (Figure 5A,B), restoring neurogenesis to levels comparable to those observed in WT controls. Notably, LY341495 had no significant effect on neurogenesis in healthy WT mice, highlighting its selective action under conditions of pathological impairment. This selective enhancement of hippocampal neurogenesis may contribute to the therapeutic effects of LY341495 in addressing cognitive deficits associated with impaired neuronal regeneration.

### 3.6 LY341495 Increased Excitatory Synaptic Activity in APP/PS1 Mice

**FIGURE 6:**
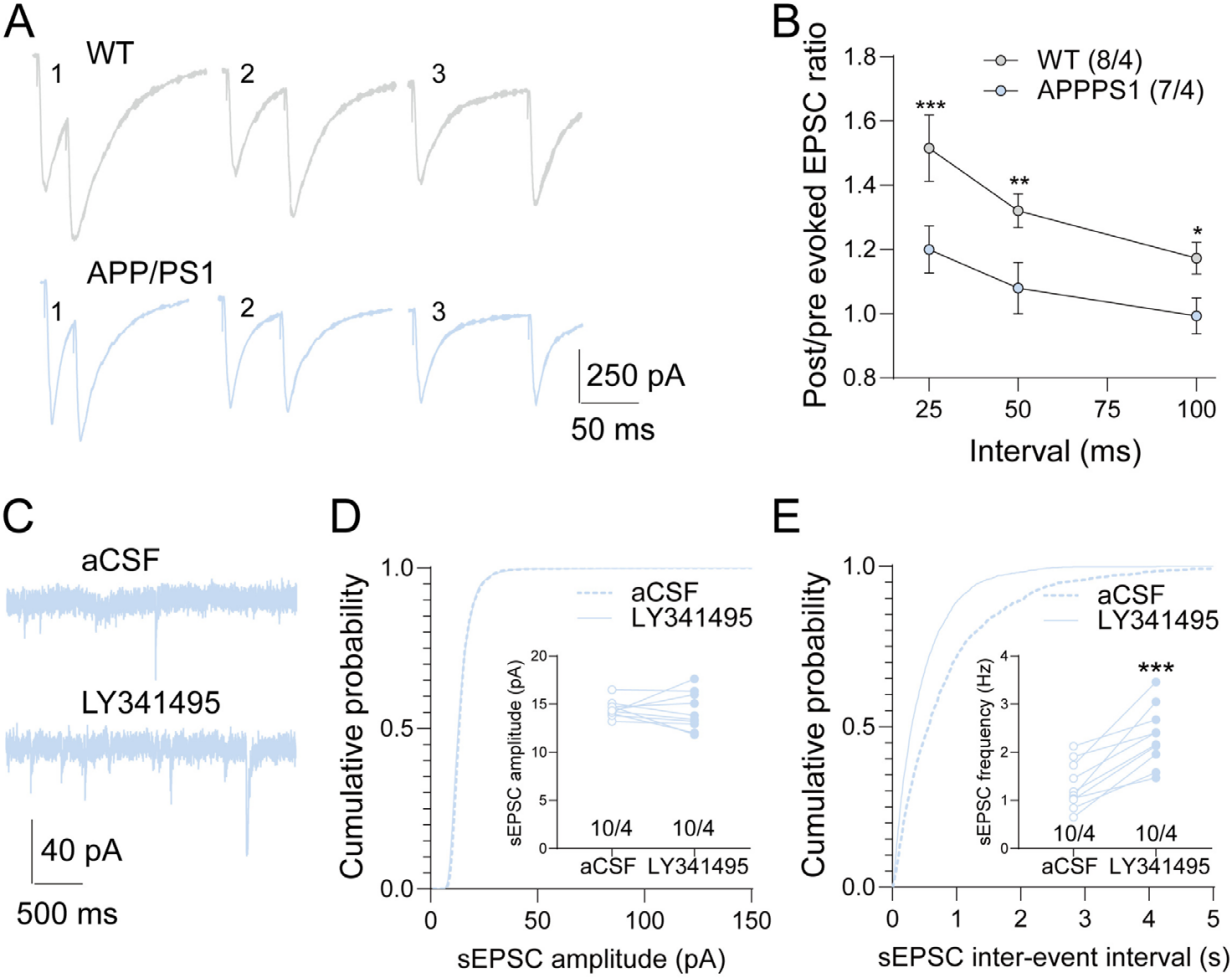
LY341495 increased excitatory synaptic activity in APP/PS1 mice. (A) Representative traces of evoked EPSCs in WT and APP/PS1 mice during paired‐pulse stimulation at 25 ms, 50 ms, and 100 ms intervals. (B) Quantification of paired‐pulse ratios (post/pre EPSC amplitude) at different intervals. APP/PS1 mice exhibited significantly reduced paired‐pulse ratio compared to WT mice at all intervals (*p < 0.05, **p < 0.01, ***p < 0.001). (C) Representative traces of spontaneous EPSCs (sEPSCs) recorded from APP/PS1 mice in the presence of aCSF or LY341495. (D) Cumulative probability plot of sEPSC amplitudes showing no significant differences between aCSF and LY341495 conditions. Inset: Mean sEPSC amplitudes for each condition. (E) Cumulative probability plot of sEPSC inter‐event intervals showing a significant reduction with LY341495 treatment. Inset: Mean sEPSC frequency significantly increased with LY341495 treatment (***p < 0.001). Data are presented as mean ± SEM. Statistical significance was determined by two‐way ANOVA or paired *t*‐tests where appropriate. *n* indicates the number of recorded neurons, and N indicates the number of mice. Both values are provided in each panel as n/N.

Previous studies have suggested that mGluR2/3 contributes to the presynaptic inhibition of glutamate signaling [7]. To evaluate synaptic function, whole‐cell electrophysiological recordings were performed on GCs in hippocampal slices from both WT and APP/PS1 mice. Paired‐pulse ratio (PPR) analysis revealed a significantly reduced post/pre EPSC ratio in APP/PS1 mice compared to WT controls at all intervals (Figure 6A,B). A reduced PPR is generally interpreted as an increase in presynaptic glutamate release probability, suggesting altered presynaptic regulation of excitatory transmission in APP/PS1 mice. We next examined spontaneous excitatory synaptic activity. Treatment with LY341495 significantly increased the frequency of spontaneous EPSCs (sEPSCs) in APP/PS1 mice, whereas sEPSC amplitudes remained unchanged (Figure 6C–E). These findings indicate that LY341495 increases excitatory synaptic activity, likely through modulation of presynaptic glutamatergic signaling, without markedly affecting postsynaptic responsiveness. Together, these results suggest that pharmacological inhibition of mGluR2/3 by LY341495 enhances synaptic transmission in the DG of APP/PS1 mice, which may contribute to the improved cognitive performance observed in behavioral tests.

## 4 Discussion

This study demonstrates that LY341495 effectively mitigates hippocampal dysfunction in APP/PS1 mice. Specifically, LY341495 treatment initiated at 6 months of age significantly improved recognition and spatial memory, reduced hippocampal plaque load, restored mGluR2/3‐mediated synaptic transmission homeostasis, and increased calbindin expression and neurogenesis in the DG. These findings highlight the potential of LY341495 as a therapeutic candidate for AD, offering a novel approach to mitigate hippocampal dysfunction.

In APP/PS1 mice, we observed a pathological upregulation of mGluR2/3 expression in hippocampal regions, accompanied by reduced glutamate release probability. These abnormalities disrupt synaptic plasticity and are closely associated with cognitive impairments, including deficits in recognition and spatial memory. mGluR2/3 are predominantly localized at presynaptic terminals, where they function as inhibitory auto‐receptors to regulate glutamate release. This regulation is crucial for maintaining synaptic transmission homeostasis and preventing excitotoxicity, a pathological condition characterized by excessive glutamate receptor activation leading to neuronal damage and death [18, 19]. Previous studies have reported that increased mGluR2/3 expression in postmortem hippocampal tissues of AD patients and in animal models of Aβ pathology. This suggests that the upregulation of mGluR2/3 may represent a compensatory response to excitatory toxicity in the AD brain [5, 20]. However, excessive activation of mGluR2/3 results in an over‐suppression of presynaptic glutamate release, leading to synaptic depression, impaired information processing, and cognitive dysfunction [21]. Our findings align with these observations and provide further evidence that Aβ pathology disrupts mGluR2/3 signaling through several mechanisms [20], including dysregulation of mGluR2/3‐mediated synaptic transmission homeostasis, reduced glutamate release probability, and impairments in calbindin expression and neurogenesis. Notably, we found that mGluR2/3 upregulation coincided with Aβ plaque deposition in the hippocampus of APP/PS1 mice, indicating a pathological feedback loop in which Aβ exacerbates mGluR2/3 dysregulation, further impairing synaptic function and contributing to cognitive decline. It is important to note that LY341495 functions as a competitive antagonist of mGluR2/3 and is not expected to directly downregulate receptor expression. Therefore, the observed attenuation of mGluR2/3 immunoreactivity in APP/PS1 mice likely reflects an indirect effect. One possible explanation is that reduced Aβ plaque burden following LY341495 treatment alleviates pathological feedback mechanisms that drive receptor upregulation. Alternatively, restoration of synaptic activity and glutamatergic homeostasis may secondarily normalize receptor expression levels. Further studies will be required to determine the precise molecular mechanisms underlying this regulation.

LY341495, a selective mGluR2/3 antagonist, demonstrated efficacy in normalizing mGluR2/3 expression in APP/PS1 mice, particularly in regions exhibiting pronounced pathological upregulation. By antagonizing mGluR2/3, LY341495 mitigated the excessive inhibitory signaling of these receptors, thereby restoring glutamatergic signaling homeostasis. This resulted in an increase in presynaptic glutamate release, enhanced synaptic transmission, and the re‐establishment of normal synaptic activity [22, 23]. Importantly, these synaptic improvements were accompanied by significant enhancements in cognitive performance, including recognition and spatial memory, as evidenced by behavioral assessments. These findings are consistent with previous studies showing that LY341495 enhances glutamate release and supports cognitive performance in animal models [7, 8]. In addition to its effects on synaptic transmission, mGluR2/3 activity has been implicated in the regulation of Aβ pathology [24]. LY341495 has been shown to inhibit these processes by suppressing mGluR2/3 activity, thereby reducing Aβ plaque load in the hippocampus [10]. This reduction in plaque load is associated with improved neuronal integrity and cognitive outcomes in preclinical models of ad [25]. Furthermore, LY341495 has been reported to block Aβ‐induced neurotoxicity in vitro and prevent the accumulation of Aβ_42_, a key component of amyloid plaques [9]. Consistent with these findings, our study demonstrated that LY341495 treatment significantly reduced hippocampal plaque deposition in APP/PS1 mice. This reduction further supports the potential of LY341495 to modulate Aβ pathology and mitigate its downstream effects on neuronal function and cognition.

However, differences exist between our findings and other reports. While some studies suggest that mGluR2/3 activation may have neuroprotective effects by reducing excitotoxicity [26, 27], our results indicate that pathological upregulation of mGluR2/3 contributes to synaptic and cognitive deficits in APP/PS1 mice. This apparent discrepancy may reflect differences in the timing and extent of mGluR2/3 dysregulation during disease progression. In early stages of AD, mGluR2/3 activation may serve a protective role, but chronic upregulation in later stages could impair synaptic function by excessively suppressing glutamate release. The ability of LY341495 to restore a functional balance of glutamatergic signaling may explain its therapeutic efficacy in our model.

This study highlights the critical role of mGluR2/3 modulation in restoring calcium‐buffering capacity, as indicated by calbindin expression, and supporting synaptic integrity. Calbindin, a calcium‐binding protein, is essential for buffering intracellular calcium levels and protecting neurons from calcium‐mediated excitotoxic damage [28, 29]. In APP/PS1 mice, reduced calbindin levels in the DG impair calcium buffering capacity and increase neuronal vulnerability [13, 15]. This dysfunction may be further exacerbated by the pathological upregulation of mGluR2/3, which suppresses presynaptic glutamate release and destabilizes synaptic homeostasis [30]. LY341495 treatment restored calbindin expression in APP/PS1 mice, particularly in hippocampal regions critical for synaptic transmission. The recovery of calbindin levels may enhance neuronal calcium‐buffering capacity and thereby support more stable intracellular calcium regulation, as calcium dysregulation is a hallmark of AD and a key contributor to synaptic and cognitive decline [31, 32]. By inhibiting mGluR2/3 activity, LY341495 may relieve excessive suppression of glutamate release, thereby facilitating synaptic activity and promoting neuronal integrity. The observed improvements in synaptic transmission and cognitive function in LY341495‐treated mice further support the therapeutic potential of targeting mGluR2/3 signaling in AD.

Impaired neurogenesis is another key feature of hippocampal dysfunction in ad [33]. In APP/PS1 mice, we observed a significant reduction in hippocampal DCX^+^ cell populations, indicating diminished neurogenesis in the DG. This decline is closely associated with cognitive deficits, as hippocampal neurogenesis is vital for memory formation and learning [3]. Treatment with LY341495 significantly enhanced hippocampal neurogenesis, evidenced by the recovery of DCX^+^ cell populations in the DG. The restoration of neurogenesis likely a key contributor to the cognitive improvements seen in behavioral tests [34]. These findings align with previous studies linking mGluR2/3 inhibition to improved synaptic plasticity and structural remodeling in neurodegenerative conditions [25, 35]. The neurogenic effects of LY341495 can be attributed to its ability to enhance glutamate release, which activates NMDA and AMPA receptor‐mediated signaling pathways [36]. This activation stimulates neurogenic cascades and increases levels of brain‐derived neurotrophic factor (BDNF), a critical regulator of neuronal growth and survival [37]. By promoting these processes, LY341495 supports hippocampal plasticity and contributes to cognitive resilience in AD models.

Limitations of this study: First, while LY341495 improved dentate gyrus function in APP/PS1 mice, its long‐term effects and safety profile remain unclear. Chronic mGluR2/3 inhibition may disrupt normal glutamate homeostasis in non‐pathological conditions. Second, this study focused primarily on the DG, leaving the effects of LY341495 on other hippocampal regions, such as CA1 and CA3, unexplored. Third, while reduced Aβ plaque load was observed, the precise mechanisms linking mGluR2/3 modulation to amyloid clearance require further investigation. Fourth, this study was conducted using male mice, as the focus was on understanding the effects of LY341495 within this specific context. However, given the known influence of sex differences in AD progression, future studies will be needed to investigate the effects of LY341495 in female mice to determine whether similar benefits are observed. Finally, the APP/PS1 model primarily reflects Aβ‐related pathology, and it remains uncertain whether these findings extend to tau‐related pathology in AD.

## 5 Conclusion

In summary, this study identifies LY341495 as a promising therapeutic candidate for AD by targeting mGluR2/3 signaling. Treatment with LY341495 was associated with improvements in hippocampal DG function, including restoration of calbindin expression, enhanced neurogenesis, and improved excitatory synaptic activity in the APP/PS1 mouse model. These findings provide valuable insights into the role of mGluR2/3 in AD pathophysiology and highlight its potential as a target for therapeutic intervention. Further studies are warranted to explore the broader applicability, long‐term efficacy, and safety of LY341495 in AD and other neurodegenerative disorders.

## Author Contributions

**Cui‐Ping Chen:** investigation, formal analysis, data curation, methodology, writing – original draft. **Ting Zhang:** investigation, formal analysis, validation. **Er‐Deng E:** investigation, formal analysis. **Tian‐Yang Xu:** formal analysis, data curation. **Qing Han:** validation, funding acquisition. **Xiang Gao:** validation, funding acquisition. **Jie Sun:** validation, funding acquisition. **Yi Huang:** validation. **Jian‐Hong Yang:** writing – review and editing. **Xiao‐Qin Zhang:** conceptualization, Funding acquisition, supervision, writing – original draft, writing – review and editing.

## Funding

This work was supported by grants from the National Natural Science Foundation of China (82201322), the Natural Science Foundation of Zhejiang Province (LY24H090001), the Ningbo Top Medical and Health Research Program (2024010317, 2022020304), the Ningbo Science and Technology Project (2023H017), and the Ningbo Clinical Research Center for Emergency and Critical Diseases (2024L003).

## Ethics Statement

The animal study protocol was approved by the Committee of Ningbo University on the Ethics of Animal Experiments (NBU20240354, December 2024).

## Conflicts of Interest

The authors declare no conflicts of interest.

## Data Availability

The data that support the findings of this study are available from the corresponding author with reasonable request.

## References

[1] M. A. Good and D. M. Bannerman , “Hippocampal Synaptic Plasticity: Integrating Memory and Anxiety Impairments in the Early Stages of Alzheimer's Disease,” Current Topics in Behavioral Neurosciences 69 (2025): 27–48. 39747797 10.1007/7854_2024_565

[2] Y. Mu and F. H. Gage , “Adult Hippocampal Neurogenesis and Its Role in Alzheimer's Disease,” Molecular Neurodegeneration 6 (2011): 85. 22192775 10.1186/1750-1326-6-85 PMC3261815

[3] J. N. Geigenmüller , A. R. Tari , U. Wisloff , and T. L. Walker , “The Relationship Between Adult Hippocampal Neurogenesis and Cognitive Impairment in Alzheimer's Disease,” Alzheimer's & Dementia 20, no. 10 (2024): 7369–7383. 10.1002/alz.14179 PMC11485317 39166771

[4] M. Wang , H. Zhang , J. Liang , J. Huang , T. Wu , and N. Chen , “Calcium Signaling Hypothesis: A Non‐Negligible Pathogenesis in Alzheimer's Disease,” Journal of Advanced Research S2090‐1232, no. 25 (2025): 26–28. 10.1016/j.jare.2025.01.007 PMC12627345 39793962

[5] A. Srivastava , B. Das , A. Y. Yao , and R. Yan , “Metabotropic Glutamate Receptors in Alzheimer's Disease Synaptic Dysfunction: Therapeutic Opportunities and Hope for the Future,” Journal of Alzheimer's Disease 78, no. 4 (2020): 1345–1361. 10.3233/JAD-201146 PMC8439550 33325389

[6] S. H. Li , K. S. Abd‐Elrahman , and S. S. G. Ferguson , “Targeting mGluR2/3 for Treatment of Neurodegenerative and Neuropsychiatric Diseases,” Pharmacology & Therapeutics 239 (2022): 108275. 36038019 10.1016/j.pharmthera.2022.108275

[7] P. S. Pinheiro and C. Mulle , “Presynaptic Glutamate Receptors: Physiological Functions and Mechanisms of Action,” Nature Reviews. Neuroscience 9, no. 6 (2008): 423–436. 18464791 10.1038/nrn2379

[8] X.‐Q. Zhang , H.‐J. Jiang , L. Xu , et al., “The Metabotropic Glutamate Receptor 2/3 Antagonist LY341495 Improves Working Memory in Adult Mice Following Juvenile Social Isolation,” Neuropharmacology 177 (2020): 108231. 32693006 10.1016/j.neuropharm.2020.108231

[9] F. Caraci , G. Molinaro , G. Battaglia , et al., “Targeting Group II Metabotropic Glutamate (mGlu) Receptors for the Treatment of Psychosis Associated With Alzheimer's Disease: Selective Activation of mGlu2 Receptors Amplifies Beta‐Amyloid Toxicity in Cultured Neurons, Whereas Dual Activation of mGlu2 and mGlu3 Receptors Is Neuroprotective,” Molecular Pharmacology 79, no. 3 (2011): 618–626. 21159998 10.1124/mol.110.067488

[10] S. H. Kim , P. E. Fraser , D. Westaway , P. H. St George‐Hyslop , M. E. Ehrlich , and S. Gandy , “Group II Metabotropic Glutamate Receptor Stimulation Triggers Production and Release of Alzheimer's Amyloid(Beta)42 From Isolated Intact Nerve Terminals,” Journal of Neuroscience 30, no. 11 (2010): 3870–3875. 20237257 10.1523/JNEUROSCI.4717-09.2010 PMC2857209

[11] L. Xu , Y. Zhou , L. Hu , et al., “Deficits in N‐Methyl‐D‐Aspartate Receptor Function and Synaptic Plasticity in Hippocampal CA1 in APP/PS1 Mouse Model of Alzheimer's Disease,” Frontiers in Aging Neuroscience 13 (2021): 772980. 34916926 10.3389/fnagi.2021.772980 PMC8669806

[12] T. M. Locke , M. E. Soden , S. M. Miller , et al., “Dopamine D1 Receptor–Positive Neurons in the Lateral Nucleus of the Cerebellum Contribute to Cognitive Behavior,” Biological Psychiatry 84, no. 6 (2018): 401–412. 29478701 10.1016/j.biopsych.2018.01.019 PMC6072628

[13] X. Zhang , Y. Mei , Y. He , et al., “Ablating Adult Neural Stem Cells Improves Synaptic and Cognitive Functions in Alzheimer Models,” Stem Cell Reports 16, no. 1 (2021): 89–105. 33382977 10.1016/j.stemcr.2020.12.003 PMC7897582

[14] G. L. Odero , K. Oikawa , K. A. Glazner , et al., “Evidence for the Involvement of Calbindin D28k in the Presenilin 1 Model of Alzheimer's Disease,” Neuroscience 169, no. 1 (2010): 532–543. 20399254 10.1016/j.neuroscience.2010.04.004

[15] S. Y. Kook , H. Jeong , M. J. Kang , et al., “Crucial Role of Calbindin‐D28k in the Pathogenesis of Alzheimer's Disease Mouse Model,” Cell Death and Differentiation 21, no. 10 (2014): 1575–1587. 24853300 10.1038/cdd.2014.67 PMC4158683

[16] M. Yamagishi , S. Takami , and T. V. Getchell , “Ontogenetic Expression of Spot 35 Protein (Calbindin‐D28k) in Human Olfactory Receptor Neurons and Its Decrease in Alzheimer's Disease Patients,” Annals of Otology, Rhinology, and Laryngology 105, no. 2 (1996): 132–139. 8659934 10.1177/000348949610500208

[17] E. Verdaguer , S. Brox , D. Petrov , et al., “Vulnerability of Calbindin, Calretinin and Parvalbumin in a Transgenic/Knock‐In APPswe/PS1dE9 Mouse Model of Alzheimer Disease Together With Disruption of Hippocampal Neurogenesis,” Experimental Gerontology 69 (2015): 176–188. 26099796 10.1016/j.exger.2015.06.013

[18] C. M. Niswender and P. J. Conn , “Metabotropic Glutamate Receptors: Physiology, Pharmacology, and Disease,” Annual Review of Pharmacology and Toxicology 50 (2010): 295–322. 10.1146/annurev.pharmtox.011008.145533 PMC2904507 20055706

[19] J. Cartmell and D. D. Schoepp , “Regulation of Neurotransmitter Release by Metabotropic Glutamate Receptors,” Journal of Neurochemistry 75, no. 3 (2000): 889–907. 10936169 10.1046/j.1471-4159.2000.0750889.x

[20] C. A. Findley , A. Bartke , K. N. Hascup , and E. R. Hascup , “Amyloid Beta‐Related Alterations to Glutamate Signaling Dynamics During Alzheimer's Disease Progression,” ASN Neuro 11 (2019): 1759091419855541. 31213067 10.1177/1759091419855541 PMC6582288

[21] H. S. Engelman and A. B. MacDermott , “Presynaptic Ionotropic Receptors and Control of Transmitter Release,” Nature Reviews. Neuroscience 5, no. 2 (2004): 135–145. 14735116 10.1038/nrn1297

[22] X. Chen , R. Lin , L. Chang , et al., “Enhancement of Long‐Term Depression by Soluble Amyloid β Protein in Rat Hippocampus Is Mediated by Metabotropic Glutamate Receptor and Involves Activation of p38MAPK, STEP and Caspase‐3,” Neuroscience 253 (2013): 435–443. 24012839 10.1016/j.neuroscience.2013.08.054

[23] S. M. Fitzjohn , Z. A. Bortolotto , M. J. Palmer , et al., “The Potent mGlu Receptor Antagonist LY341495 Identifies Roles for Both Cloned and Novel mGlu Receptors in Hippocampal Synaptic Plasticity,” Neuropharmacology 37, no. 12 (1998): 1445–1458. 9886667 10.1016/s0028-3908(98)00145-2

[24] J. R. Cirrito , K. A. Yamada , M. B. Finn , et al., “Synaptic Activity Regulates Interstitial Fluid Amyloid‐Beta Levels In Vivo,” Neuron 48, no. 6 (2005): 913–922. 16364896 10.1016/j.neuron.2005.10.028

[25] S. H. Kim , J. W. Steele , S. W. Lee , et al., “Proneurogenic Group II mGluR Antagonist Improves Learning and Reduces Anxiety in Alzheimer Aβ Oligomer Mouse,” Molecular Psychiatry 19, no. 11 (2014): 1235–1242. 25113378 10.1038/mp.2014.87 PMC4217144

[26] V. N. Bukke , M. Archana , R. Villani , et al., “The Dual Role of Glutamatergic Neurotransmission in Alzheimer's Disease: From Pathophysiology to Pharmacotherapy,” International Journal of Molecular Sciences 21, no. 20 (2020): 7452. 33050345 10.3390/ijms21207452 PMC7589203

[27] S. F. Sonnenschein and A. A. Grace , “Peripubertal mGluR2/3 Agonist Treatment Prevents Hippocampal Dysfunction and Dopamine System Hyperactivity in Adulthood in MAM Model of Schizophrenia,” Schizophrenia Bulletin 47, no. 6 (2021): 1806–1814. 33928393 10.1093/schbul/sbab047 PMC8530391

[28] G. L. Rintoul , L. A. Raymond , and K. G. Baimbridge , “Calcium Buffering and Protection From Excitotoxic Cell Death by Exogenous Calbindin‐D28k in HEK 293 Cells,” Cell Calcium 29, no. 4 (2001): 277–287. 11243935 10.1054/ceca.2000.0190

[29] M. P. Mattson , B. Rychlik , C. Chu , and S. Christakos , “Evidence for Calcium‐Reducing and Excito‐Protective Roles for the Calcium‐Binding Protein Calbindin‐D28k in Cultured Hippocampal Neurons,” Neuron 6, no. 1 (1991): 41–51. 1670921 10.1016/0896-6273(91)90120-o

[30] M. A. Kim and C. J. Jeon , “Metabotropic Glutamate Receptor mGluR2/3 Immunoreactivity in the Mouse Superior Colliculus: Co‐Localization With Calbindin D28K,” Neuroreport 10, no. 6 (1999): 1341–1346. 10363950 10.1097/00001756-199904260-00034

[31] L. Song , Y. Tang , and B. Y. K. Law , “Targeting Calcium Signaling in Alzheimer's Disease: Challenges and Promising Therapeutic Avenues,” Neural Regeneration Research 19, no. 3 (2024): 501–502. 37721273 10.4103/1673-5374.380898 PMC10581553

[32] J. J. Palop , B. Jones , L. Kekonius , et al., “Neuronal Depletion of Calcium‐Dependent Proteins in the Dentate Gyrus Is Tightly Linked to Alzheimer's Disease‐Related Cognitive Deficits,” Proceedings of the National Academy of Sciences of the United States of America 100, no. 16 (2003): 9572–9577. 12881482 10.1073/pnas.1133381100 PMC170959

[33] L. Ma , Q. Wei , M. Jiang , et al., “Hippocampal Neurogenesis in Alzheimer's Disease: Multimodal Therapeutics and the Neurogenic Impairment Index Framework,” International Journal of Molecular Sciences 26, no. 13 (2025): 6105. 40649882 10.3390/ijms26136105 PMC12250571

[34] M. Mostafa , A. Disouky , and O. Lazarov , “Therapeutic Modulation of Neurogenesis to Improve Hippocampal Plasticity and Cognition in Aging and Alzheimer's Disease,” Neurotherapeutics 22, no. 3 (2025): e00580. 40180804 10.1016/j.neurot.2025.e00580 PMC12047516

[35] G. Perez Garcia , M. Bicak , J. Buros , et al., “Beneficial Effects of Physical Exercise and an Orally Active mGluR2/3 Antagonist Pro‐Drug on Neurogenesis and Behavior in an Alzheimer's Amyloidosis Model,” Front Dement 2 (2023): 1198006. 39081972 10.3389/frdem.2023.1198006 PMC11285632

[36] L. Deutschenbaur , J. Beck , A. Kiyhankhadiv , et al., “Role of Calcium, Glutamate and NMDA in Major Depression and Therapeutic Application,” Progress in Neuro‐Psychopharmacology & Biological Psychiatry 64 (2016): 325–333. 25747801 10.1016/j.pnpbp.2015.02.015

[37] T. Numakawa , H. Odaka , and N. Adachi , “Actions of Brain‐Derived Neurotrophin Factor in the Neurogenesis and Neuronal Function, and Its Involvement in the Pathophysiology of Brain Diseases,” International Journal of Molecular Sciences 19, no. 11 (2018): 3650. 30463271 10.3390/ijms19113650 PMC6274766

